# Knowledge and Attitudes of Medical Students toward COVID-19 Vaccine in Saudi Arabia

**DOI:** 10.3390/vaccines10040541

**Published:** 2022-03-31

**Authors:** Syed Shahid Habib, Musab Saleh Alamri, Mudafr Mahmoud Alkhedr, Mohammad Abdullah Alkhorijah, Rayan Dhafer Jabaan, Mubarak Khalid Alanzi

**Affiliations:** 1 Department of Physiology, College of Medicine, King Saud University, Riyadh 11461, Saudi Arabia; 2 College of Medicine, King Saud University, Riyadh 11461, Saudi Arabia; 439100638@student.ksu.edu.sa (M.S.A.); 439105282@student.ksu.edu.sa (M.M.A.); 439100540@student.ksu.edu.sa (M.A.A.); 439100696@student.ksu.edu.sa (R.D.J.); 439100719@student.ksu.edu.sa (M.K.A.)

**Keywords:** knowledge, attitudes, COVID-19 vaccine, medical students, Saudi Arabia

## Abstract

Medical students are the future caregivers of communities, and therefore it is important to rectify their misconceptions about the COVID-19 vaccine. We aimed to explore the knowledge and attitudes among medical students toward the COVID-19 vaccine in Saudi Arabia and to compare the level of knowledge between preclinical and clinical years. This epidemiological cross-sectional study of 1445 (47.3% were pre-clinical and 52.7% were clinical year) medical students was conducted at various universities in Saudi Arabia using a simple random sampling technique. The results revealed that 34.3% students did not know how the Pfizer vaccine worked, with a high proportion in preclinical students (69.4%). Almost 37% of participants thought that one could become infected with COVID-19 via the COVID-19 vaccine, and 67.1% of these students were pre-clinical. About 22.6% of students did not trust COVID-19 vaccine information from the health ministry, and the majority of them (79.8%) were pre-clinical. Vaccine hesitancy was shown by about 33.3% (*n* = 481) of subjects, and surprisingly, almost half of them (48.6%) thought that the COVID-19 vaccine involved conspiracy; the majority of them were pre-clinical (97.9%). The overall response of students indicates a significantly lower level of knowledge and increased negative attitudes of preclinical students toward the COVID-19 vaccine. However, the vast majority of students agreed on the importance of the COVID-19 vaccine to decrease the spread of the disease.

## 1. Introduction

COVID-19 is a highly contagious coronavirus disease caused by the recently discovered severe acute respiratory syndrome CoV-2 (SARS-CoV-2), which was first discovered in Wuhan, China [1]. It was considered a global pandemic by the end of January 2020. Its clinical manifestation varies, but the most common symptoms are fever or chills, cough, headache, and loss of taste or smell; it can be asymptomatic (patients who never have symptoms) [2]. As of 9 March 2022, 450,675,294 cases and 6,039,087 deaths have been reported globally [3]. On 31 December 2020, the World Health Organization (WHO) announced that the mRNA vaccine was acceptable for emergency use, making the Pfizer/BioNTech vaccine the first to receive emergency validation from the WHO since the outbreak began [4]. Other vaccinations have also been approved, and these vaccines have the potential to put an end to the pandemic. In addition, several vaccines are currently undergoing clinical assessments. A number of them have received Emergency Use Authorization (EUA) from the US Food and Drug Administration (US FDA) and are now utilized in a variety of countries (e.g., Pfizer BioNTech, Moderna, and Janssen vaccines) [5,6]. Moreover, the Saudi Food and Drug Authority (SFDA) approved the registration of the coronavirus vaccines from Pfizer-BioNTech, AstraZeneca-Oxford, and Moderna [7]. The necessity for vaccination is rising by the day, with increasing numbers of cases of virus variations in several countries. These vaccines may bring an end to this pandemic.

Vaccine hesitancy is one of the ten main dangers to world health, according to the World Health Organization (WHO) [8]. Asking the public to take the COVID-19 vaccine is a sensitive topic for many populations. Convincing people to get the COVID-19 vaccine depends on multiple factors, such misconceptions, lack of literacy, lack of trust in the health system, and vaccine safety and efficacy [9,10,11]. Furthermore, it is important to achieve high vaccination rates among medical students, because they are more likely to face COVID-19 in their practice and they need to counsel hesitant individuals as the next generation of doctors [12]. A study conducted by Lucia on medical students (*n* = 167) in Southeast Michigan showed that 98% believed that finding a COVID-19 vaccination is critical for reducing community spread. Even though 98% of students believed that they would most likely be exposed to COVID-19, 23% said they would not take a COVID-19 vaccine immediately after FDA approval [12]. Another study on medical students in two universities in Egypt (*n* = 2133) showed that the most reported barriers to COVID-19 vaccination were insufficient information regarding the adverse effect of the vaccine (74.4%) and insufficient information regarding the vaccine itself (72.8%) [13]. According to Lucia, the medical students’ concerns about the serious side effects of the COVID-19 vaccine were intimately associated with their confidence in the information they were receiving regarding the COVID-19 vaccine. Only 87% of the medical students sampled believed that public health experts are trustworthy when it comes to the information that they provide about the COVID-19 vaccine [12].

In Saudi Arabia, immunization is an obligatory requirement, as of August 2021, for entry into government, private, and educational activities, events, and facilities; use of public transportation; and the return of in-person education for teachers and faculty members in universities and training institutions [14]. Therefore, medical students cannot enter university facilities unless they have all required COVID-19 vaccine doses. Moreover, free education, the cultural environment, and deep religiosity make Saudi students unique in the Gulf region.

There is a paucity of data about the Coronavirus vaccine among medical students regarding knowledge and attitudes. Therefore, we aimed to explore the knowledge and attitudes among medical students regarding the COVID-19 vaccine in Saudi Arabia and to compare the level of knowledge about the COVID-19 vaccine among preclinical and clinical medical students.

## 2. Materials and Methods

### 2.1. Study Design, Population, and Sample

Our study used a cross-sectional design. The targeted population consisted of medical students from multiple universities in Saudi Arabia from August 2021 to October 2021, Using Epi Info version 7.2.4 (Center for Disease Control and Prevention, Atlanta, GA, USA) [15], the ideal sample size was estimated based on the population size (20,840) [16]; we used a 50% anticipated frequency, 5% margin of error, and 99% confidence interval, which was calculated to be 1030 participants, and we added 15% to account for the non-responders or incomplete responses. Therefore, the total sample was around 1185.

### 2.2. Questionnaire

A questionnaire was developed for this study, which consisted of three parts: socio-demographics, and questions about knowledge of and attitudes toward the COVID-19 vaccine. The criteria for choosing the questions were based on the literature search, our previous research study, and a large-scale survey conducted by the Saudi Ministry of Health for Saudi residents [17,18]. We included those items that were not previously reported or were of specific interest. The first part of the questionnaire included the socio-demographic variables of gender, age, nationality, place of residence, university, current year, family income, which vaccine they had taken, and whether they had been infected with COVID-19 or not. The trichotomous variables with (Yes/No/I don’t know) answers were coded 1 for yes, 2 for no, and 3 for I don’t know. The demographic variables were coded as follows: gender (1 for male and 2 for female), current year (1 for pre-clinical and 2 for clinical).

The second part of the questionnaire (knowledge about COVID-19 vaccine) consisted of eight main questions on the participants’ knowledge of COVID-19 vaccines that have been used in Saudi Arabia. We asked the participants different questions related to the COVID-19 vaccine, with six items having options “Yes/No/I don’t know” and two questions with multiple answers related to the mechanism of action of the COVID vaccine and the sources of information. The knowledge was compared between clinical and pre-clinical medical students.

The third part of the questionnaire (attitudes toward COVID-19 vaccine) consisted of thirteen main questions on the participants’ attitudes toward the COVID-19 vaccine, which included eleven items as “Yes/No/I don’t know” and two additional questions. The first regarded the participants’ opinion on the most appropriate way to deal with the COVID-19 vaccine, and the second sought the participants’ reasons for refusing or being hesitant about the COVID-19 vaccine.

The questionnaire was tested on a group of university medical students to ensure its clarity before distribution.

### 2.3. Data Collection 

An online survey using a Google form was conducted between 20 August 2021 and 6 October 2021. A simple random technique was used to recruit study participants. It was done via contacting leaders of different areas from multiple universities and asking for contact information of students and then arranging the information in a list from which we chose participants randomly. We distributed the questionnaire through informal channels such as direct messages on social media like WhatsApp (WhatsApp Inc., Mountain View, CA, USA), e-mail, or text messages.

### 2.4. Ethical Consideration

Ethical clearance was obtained from the Institutional Review Board of the College of Medicine, King Saud University, Saudi Arabia (E-21-6165/CMED-305/A9E-19-4404). Although participation was voluntary, all participants were asked for their agreement before accessing the questionnaire items via a yes/no question about their willingness to participate in the study. In addition, participants were also given all necessary information on the target population, as well as instructions for filling out the questionnaire and the confidentiality procedures applied to preserve the obtained data.

### 2.5. Statistical Analysis

Data were extracted from Google Forms (Google Inc, Mountain View, CA, USA) to an Excel sheet (Microsoft, Redmond, WA, USA) and statistically analyzed using IBM SPSS software version 21 (SPSS Inc, IL, USA). Qualitative data were presented as frequencies and percentages. The chi-square test was used to assess the association between two categorical variables. For different categories of knowledge and attitude, a comparison was made between pre-clinical and clinical students. A *p*-value of < 0.05 was used to report the statistical significance.

## 3. Results

### 3.1. Sociodemographic Characteristics of Participants

The total number of participants was 1747. There were 302 non-respondents, which represented 17.28% of the total; thus, the final population included in this study was 1445, which is a representative sample size according to our power calculations, with a predictive power of more than 80%. Since the non-responders were in equal proportions from various universities, it did not affect the results. About 89.3% (*n* = 1291) of students participated from the central region of Saudi Arabia. The majority of the participants, 88.7% (*n* = 1282), were males, while 98.8% (*n* = 1428) of the sample were Saudis. Over half of the participants, 55.5% (*n* = 802), were studying at King Saud University, and all of the participants were divided into pre-clinical years (which represents the first two years of medical school) (47.3%, *n* = 683), and clinical years (which represents the remaining three years of medical school) (52.7%, *n* = 762). Approximately two-thirds of the participants reported a family income of >SR 20,000 a month (64.8%, *n* = 937). The number of participants who were vaccinated by BioNTech/Pfizer was 625 (43.3%). Over three-quarters of participants reported that they had never been infected with COVID-19 (79.6%, *n* = 1150) (Table 1).

### 3.2. Knowledge Related to the COVID-19 Vaccine

Data concerning knowledge related to the COVID-19 vaccine and sources of information showed that 18% (*n* = 260) of participants, 91.5% (*n* = 238) of whom were pre-clinical students, thought that the COVID-19 vaccine was not important in decreasing community spread. More than one-third of medical students (34.3%, *n* = 496) (*p* < 0.001 for all) stated that they did not know how the Pfizer vaccine worked. Most of the medical students agreed on the safety of taking two different COVID-19 vaccines from different companies (63.6%, *n* = 919) (*p* < 0.001, for all). More than one-third of medical students stated that you can be infected with COVID-19 via the COVID-19 vaccine (37%, *n* = 535) (*p* < 0.001 for all) of which 67.1% (*n* = 359) were pre-clinical students. About 42.1% (*n* = 609) of the participants (*p* < 0.001 for all) stated that the COVID-19 vaccine prevents the spread of COVID-19. More than one-quarter of medical students (28.4%, *n* = 411) (*p* < 0.001 for all) stated that they did not know whether the COVID-19 vaccine decreases immunity. About 67.3% (*n* = 973) of respondents replied correctly that COVID-19 vaccine does not decrease immunity. Clinical students’ correct responses were significantly higher (64.6%, *n* = 628) compared to pre-clinical students (35.5%, *n* = 345). Two-thirds of medical students (66.8%, *n* = 965) (*p* < 0.001 for all) agreed that there are individuals who got COVID-19 after being fully vaccinated. Most medical students chose the Ministry of Health as their main source of information about the COVID-19 vaccine (68.92%, *n* = 996) (*p* < 0.001); the second most essential source was social media (53.2%, *n* = 769) (*p* < 0.001), followed by the WHO (World Health Organization) as third most important source of information for medical students (52.3%, *n* = 756) (*p* < 0.001) (Table 2).

### 3.3. Attitude Related to the COVID-19 Vaccine

Nearly half (48.9%, *n* = 707) (*p* < 0.001 for all) of the medical students were concerned about the safety of the COVID-19 vaccine. More than half of the respondents reported concerns about the efficacy of the COVID-19 vaccine (52.6%, *n* = 760) (*p* < 0.001 for all). About 54.7% (*n* = 790) (*p* < 0.001 for all) of medical students stated that they did not know if the COVID-19 vaccine should be taken annually. Most medical students (70.9%, *n* = 1024) reported that they trust the Ministry of Health when it comes to information about the COVID-19 vaccine. Roughly one-third of respondents stated that they thought that the COVID-19 vaccine should be a free choice to be taken (31.4%, *n* = 454) (*p* < 0.001 for all). Around two-thirds of respondents (65%, *n* = 940) (*p* < 0.001 for all) agreed with the government’s decision on making the COVID-19 vaccine a requirement to enter a university facility. About a quarter of medical students (23.5%, *n* = 340) (*p* < 0.001 for all) thought that the rapid development of the COVID-19 vaccine did not play a role in the refusal or hesitancy of the population. Less than one-half of the respondents (40.1%, *n* = 580) (*p* < 0.001 for all) would wear a mask even if the government canceled the precautions. Half of the medical students (50%, *n* = 723) (*p* < 0.001 for all) thought that the people would not take the COVID-19 vaccine if it cost money. More than one-quarter of respondents (27.9%, *n* = 403) (*p* < 0.001 for all) felt anxious about the long-term side effects of the COVID-19 vaccine. About 41.4% (*n* = 598) (*p* < 0.001 for all) thought that the COVID-19 vaccine would not return life to what it was before the pandemic. More than one-quarter of medical students (26.6%, *n* = 384) (*p* < 0.001 for all) would not take the COVID-19 vaccine if it was not a requirement to enter a university facility (Table 3).

**Figure 1:**
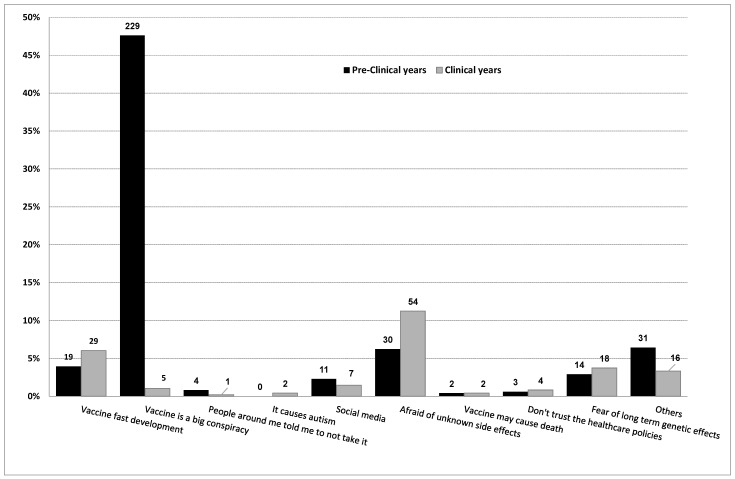
Reasons explaining negative attitudes toward COVID-19 vaccination for participants who were hesitant to be vaccinated.

Data concerning reasons behind negative attitudes of participants who would not take the COVID-19 vaccine willingly or were hesitant included about 33.3% (*n* = 481) of subjects. Approximately half of these participants (48.6%, *n* = 234) stated that the COVID-19 vaccine involves a conspiracy, and the majority of them (97.9%, *n* = 229) were pre-clinical students (Figure 1).

## 4. Discussion

In this study, we explored the knowledge and attitudes of 1445 participants toward the COVID-19 vaccine in multiple universities in Saudi Arabia, covering various years of medical school. Of these, 47.3% (*n* = 683) were pre-clinical students and 52.7% (*n* = 762) were clinical students. Of the medical students, 79.1% (*n* = 1143) agreed on the importance of developing a COVID-19 vaccine to decrease its community spread, which is lower than what has been reported in another study, which demonstrated that 98% agreed on the importance of developing a COVID-19 vaccine [12]. This decline may be due to the increase of COVID-19 variants that appeared in the months that followed the publication of the previous research, thus making medical students less certain about the effectiveness of the COVID-19 vaccine as some COVID-19 vaccines were less efficient against COVID-19 variants [19]. Quiet surprisingly, we noticed a lack of knowledge among pre-clinical students about the mechanism of action of the Pfizer-BioNTech COVID-19 vaccine, which is the most used vaccine among medical students in Saudi Arabia, based on our findings. A previous experimental study showed that “recipients of both the homologous vector (Oxford-AstraZeneca) regimen and the heterologous vector/mRNA combination reported greater reactogenicity following the priming vector vaccination. Whereas heterologous boosting was well tolerated and comparable to homologous mRNA (Pfizer-BioNTech) boosting. Taken together, the heterologous vector/mRNA boosting induces strong humoral and cellular immune responses with acceptable reactogenicity profiles” [20]. As we noticed in our study, this shows that most medical students think that it is safe to take two different vaccines from different companies.

According to the CDC (Centers for Disease Control and Prevention), “none of the approved COVID-19 vaccinations in the United States contain the live viruses that cause COVID-19, which implies that the vaccine cannot make you sick with COVID-19” [21]. Despite this, an alarming number of medical students think that they can be infected with COVID-19 via the COVID-19 vaccine. This could be the result of medical students’ dependency on unreliable social media as their second main source of information, based on our results. Expectedly, a high number of clinical students think that the vaccine does not decrease immunity. On the other hand, surprisingly, many preclinical students responded wrongly to the question that the COVID-19 vaccine decreases immunity or not, which indicates that pre-clinical students lack proper knowledge; this might be due to obtaining information from improper and untrustworthy sources. Most of the clinical students think that fully vaccinated individuals can be infected with COVID-19; in contrast, approximately half of the pre-clinical students do not know whether fully vaccinated individuals can be infected with COVID-19. Since clinical students are more exposed to COVID-19 cases in hospitals, this gives them an advantage over pre-clinical students in understanding breakthrough cases (becoming infected with COVID-19 after being fully vaccinated). A previous study reported heightened vaccination apprehension was linked to thoughts that vaccines aren’t safe or effective, as well as increased concerns regarding the quick development of the COVID-19 vaccines [22]. As expected, the majority of medical students have concerns about the safety and efficacy of the COVID-19 vaccine, and this could be attributed to the rapid development of the COVID-19 vaccines. Nevertheless, a previous study conducted in Romania showed that the majority of medical students trust the safety and efficacy of COVID-19 vaccines [23]. In our study, the vast majority of medical students trust the Ministry of Health when it comes to information about the COVID-19 vaccine; however, a minority of medical students, most of them pre-clinical students, don’t trust the Ministry of Health. In addition, most of the medical students concur with the government’s decision of making the COVID-19 vaccine a requirement in transportation and workplaces, which aligns with their choice in the previous statement. This shows that medical students trust the decisions made by the Ministry of Health during the COVID-19 pandemic. We found that most of the medical students think that the accelerated development of the COVID-19 vaccine plays a role in the hesitancy or refusal of the population because people think that the vaccine companies rushed the production of the COVID-19 vaccine and skipped a few steps, which might have compromised the safety and effectiveness of the vaccine [24]. Unexpectedly, we noticed that more than a quarter of medical students (26.6%, *n* = 384) stated that they would not take the COVID-19 vaccine if it was not a requirement to enter a university facility, which is high compared to previous studies [13,23,25]. Based on our findings, among medical students who are hesitant to be vaccinated, we noticed that the main reason for hesitancy is that they believe that the COVID-19 vaccine is part of a conspiracy, which differs from previous studies [12,13,23,25]. A previous study showed that online health information is frequently bolstered by rumors and conspiracy theories, which are not based on solid data [26]. Social media and online foreign disinformation campaigns on vaccination rates and attitudes towards vaccine safety are the main factors behind misconceptions regarding COVID-19 vaccination [27,28]. Our results were in agreement with these observations.

## 5. Conclusions

The overall response of medical students in this study indicates significantly low knowledge and substantially negative attitudes towards the COVID-19 vaccine among pre-clinical students. However, it also shows greater knowledge and more positive attitudes among clinical students. The vast majority of medical students agreed on the importance of developing and implementing the COVID-19 vaccine to decrease its community spread. The majority of pre-clinical students were hesitant about taking the COVID-19 vaccine and took it unwillingly as a university requirement because they thought the COVID-19 vaccine is a conspiracy. Our results can be used for public health promotion intervention, especially for pre-clinical students. There should be seminars, lectures, and educational videos that target pre-clinical students to counter their fears and misconceptions about the COVID-19 vaccine.

We recommend further research to be done to understand the factors that made pre-clinical students have negative attitudes toward and poor knowledge of the COVID-19 vaccine.

## 6. Strength and Limitations

Our study is one of the few studies conducted in Saudi Arabia to assess the knowledge and attitudes of medical students toward the COVID-19 vaccine; we performed probability sampling by doing simple random sampling. In terms of limitations, most of the study participants were from the central region, we could not reach private medical schools, and we had a small number of female participants.

## Figures and Tables

**Table 1:** Sociodemographic characteristics of participants.

Variables	*n* = 1445 *n* (%)
Gender	
Male	1282 (88.7)
Female	163 (11.3)
Age	
18–20	465 (32.2)
>20	980 (67.8)
Nationality	
Saudi	1428 (98.8)
Non-Saudi	17 (1.2)
Place of residence	
Central region	1291 (89.3)
Others	154 (10.7)
Your university	
King Saud University	802 (55.5)
King Saud Bin Abdulaziz University for health science	316 (21.9)
Imam Mohammad Bin Saud Islamic University	282 (19.5)
Others	45 (3.1)
Current year	
Pre-Clinical Years	683 (47.3)
Clinical Years	762 (52.7)
Family income	
<5000	43 (3)
5000–20,000	465 (32.2)
>20,000	937 (64.8)
Which vaccine did you receive?	
I didn’t get the vaccine	231 (16)
BioNTech/Pfizer and Oxford-AstraZeneca	376 (26)
BioNTech/Pfizer	625 (43.3)
Oxford-AstraZeneca	189 (13.1)
Others	24 (1.7)
Have you ever been infected with COVID-19?	
Yes	217 (15)
No	1150 (79.6)
I don’t know	78 (5.4)

**Table 2:** Knowledge related to the COVID-19 vaccine and sources of information by pre-clinical/clinical status.

Question	Pre-Clinical (*n* = 683) *n* (%)	Clinical (*n* = 762) *n* (%)	Total (*n* = 1445) *n* (%)	*p*-Value	(95% CI)
Do you agree on the importance of developing a COVID-19 vaccine to decrease its community spread?				<0.001	(1.57–1.68)
Yes	420 (36.7)	723 (63.3)	1143 (79.1)		
No	238 (91.5)	22 (8.5)	260 (18)		
I don’t know	25 (59.5)	17 (40.5)	42 (2.9)		
How does the Pfizer vaccine work?				<0.001	(2.89–3.1)
Encapsulated mRNA vaccine	268 (32.6)	553 (67.4)	821 (56.8)		
Virus-like particle vaccine	20 (38.5)	32 (61.5)	52 (3.6)		
Inactivated virus vaccine	51 (67.1)	25 (32.9)	76 (5.3)		
I don’t know	344 (69.4)	152 (30.6)	496 (34.3)		
Is it safe to take two different COVID-19 vaccines from different companies/brands?				<0.001	(1.8–1.9)
Yes	338 (36.8)	581 (63.2)	919 (63.6)		
No	256 (86.2)	41 (13.8)	297 (20.6)		
I don’t know	89 (38.9)	140 (61.1)	229 (15.8)		
Can you be infected with COVID-19 via the COVID-19 vaccine?				<0.001	(1.4–1.5)
Yes	359 (67.1)	176 (32.9)	535 (37)		
No	247 (33.5)	490 (66.5)	737 (51)		
I don’t know	77 (44.5)	96 (55.5)	173 (12)		
Does the COVID-19 vaccine prevent you from spreading COVID-19?				<0.001	(1.88–1.9)
Yes	215 (35.3)	394 (64.7)	609 (42.1)		
No	394 (57.7)	289 (42.3)	683 (47.3)		
I don’t know	74 (48.4)	79 (51.6)	153 (10.6)		
Does the COVID-19 vaccine decrease your immunity?				<0.001	(2.5–2.7)
Yes	35 (57.4)	26 (42.6)	61 (4.2)		
No	345 (35.5)	628 (64.5)	973 (67.3)		
I don’t know	303 (73.7)	108 (26.3)	411 (28.4)		
Has anyone gotten COVID-19 after being fully vaccinated?				<0.001	(2.1–2.3)
Yes	335 (34.7)	630 (65.3)	965 (66.8)		
No	28 (49.1)	29 (50.9)	57 (3.9)		
I don’t know	320 (75.7)	103 (24.3)	423 (29.3)		
What are your sources of information about the COVID-19 vaccine? *					
Social media	292 (38)	477 (62)	769 (53.2)	<0.001	(0.3–0.4)
People around you/Friends	177 (37.1)	300 (62.9)	477 (33.01)	<0.001	(0.20–0.28)
WHO (World Health Organisation)	244 (32.3)	512 (67.7)	756 (52.3)	<0.001	(0.21–0.29)
Ministry of health	355 (35.6)	641 (64.4)	996 (68.92)	<0.001	(0.31–0.39)
Radio	235 (87.4)	34 (12.6)	269 (18.61)	<0.001	(0.44–0.53)
Television	96 (39)	150 (61)	246 (17.02)	0.004	(0.06–0.12)
Newspaper	1 (25)	3 (75)	4 (0.27)	0.372	(−0.002–0.006)
Others	8 (22.9)	27 (77.1)	35 (2.422)	0.003	(0.009–0.04)

* Multiple answers allowed, ≠100%.

**Table 3:** Attitude related to COVID-19 vaccine and government’s decisions by pre-clinical/clinical status.

Question	Pre-Clinical (*n* = 683) *n* (%)	Clinical (*n* = 762) *n* (%)	Total (*n* = 1445) *n* (%)	*p*-Value	(95% CI)
Are you concerned about the safety of the COVID-19 vaccine?				<0.001	(1.23–1.34)
Yes	410 (58)	297 (42)	707 (48.9)		
No	236 (36.3)	414 (63.7)	650 (45)		
I don’t know	37 (42)	51 (58)	88 (6.1)		
Are you concerned about the efficacy of the COVID-19 vaccine?				<0.001	(1.22–1.32)
Yes	432 (56.8)	328 (43.2)	760 (52.6)		
No	219 (35.7)	394 (64.3)	613 (42.4)		
I don’t know	32 (44.4)	40 (55.6)	72 (5)		
Should the COVID-19 vaccine be taken annually?				<0.001	(2.56–2.68)
Yes	87 (39.4)	134 (60.6)	221 (15.3)		
No	147 (33.9)	287 (66.1)	434 (30)		
I don’t know	449 (56.8)	341 (43.2)	790 (54.7)		
Do you trust the Ministry of Health when it comes to information about the COVID-19 vaccine?				<0.001	(1.8–1.9)
Yes	382 (37.3)	642 (62.7)	1024 (70.9)		
No	261 (79.8)	66 (20.2)	327 (22.6)		
I don’t know	40 (42.6)	54 (57.4)	94 (6.5)		
Do you agree with the government’s decision about the COVID-19 vaccine being a requirement to enter a university facility?				<0.001	(1.85–1.96)
Yes	350 (37.2)	590 (62.8)	940 (65)		
No	273 (70.5)	114 (29.5)	387 (26.8)		
I don’t know	60 (50.8)	58 (49.2)	118 (8.2)		
In your opinion, what is the best way to deal with the vaccine?				<0.001	(1.76–2.02)
Free choice to take the vaccine or not	309 (68.1)	145 (31.9)	454 (31.4)		
Imposing vaccination on specific groups of people	38 (39.2)	59 (60.8)	97 (6.7)		
Make it mandatory for everyone	99 (35.4)	181 (64.6)	280 (19.4)		
Make it a requirement in transportation and workplace	168 (36.8)	289 (63.2)	457 (31.6)		
I am not sure	69 (43.9)	88 (56.1)	157 (10.9)		
Does the rapid development of the COVID-19 vaccine play any role in the refusal or hesitancy of the population?				<0.001	(1.73–1.85)
Yes	285 (33.2)	574 (66.8)	859 (59.4)		
No	268 (78.8)	72 (21.2)	340 (23.5)		
I don’t know	130 (52.8)	116 (47.2)	246 (17)		
If the government canceled the precautions, would you wear the mask even though you received a vaccine?				<0.001	(1.88–1.98)
Yes	214 (36.9)	366 (63.1)	580 (40.1)		
No	393 (56.6)	301 (43.4)	694 (48)		
I don’t know	76 (44.4)	95 (55.6)	171 (11.8)		
Do you think people would take the COVID-19 vaccine even if it costs money?				<0.001	(1.98–2.08)
Yes	174 (38.5)	278 (61.5)	452 (31.3)		
No	389 (53.8)	334 (46.2)	723 (50)		
I don’t know	120 (44.4)	150 (55.6)	270 (18.7)		
Do you feel anxious about the long-term side effects of the COVID-19 vaccine?				<0.001	(1.79–1.90)
Yes	159 (39.5)	244 (60.5)	403 (27.9)		
No	452 (51.5)	425 (48.5)	877 (60.7)		
I don’t know	72 (43.6)	93 (56.4)	165 (11.4)		
Do you think the COVID-19 vaccine will return life to what it was before the pandemic?				<0.001	(2.02–2.12)
Yes	215 (38.7)	341 (61.3)	556 (38.5)		
No	353 (59)	245 (41)	598 (41.1)		
I don’t know	115 (39.5)	176 (60.5)	291 (20.1)		
COVID-19 vaccine is a requirement to enter a university facility; if it wasn’t a requirement, would you still take the COVID-19 vaccine?				<0.001	(2.16–2.23)
Yes	340 (35.3)	624 (64.7)	964 (66.7)		
No	293 (76.3)	91 (23.7)	384 (26.6)		
I don’t know	50 (51.5)	47 (48.5)	97 (6.7)		

## Data Availability

The data presented in this study are available on request from the corresponding author.
